# Low-Power Magnetic Displacement Sensor Based on RISC-V Embedded System

**DOI:** 10.3390/s24134224

**Published:** 2024-06-29

**Authors:** Tao Sun, Yue Song, Huiyun Yang

**Affiliations:** Academy of Intelligent Innovation, Shandong Provincial Key Laboratory of New Generation Semiconductor Technology and System, Shandong University, Jinan 250101, China; 202332357@mail.sdu.edu.cn (Y.S.); 202000201029@mail.sdu.edu.cn (H.Y.)

**Keywords:** risc-v, displacement sensor, magnetic flux

## Abstract

With the emergence of RISC-V architecture in embedded devices, its inherent low-power features have propelled its extensive adoption across various industrial settings. Displacement sensors leveraging Hall sensors and magnetic flux measurement present notable benefits including cost-effectiveness and compact design. This study undertakes the porting of Hall sensors onto RISC-V architecture embedded devices, validating their functionality within displacement sensors. Empirical investigations substantiate that the ported system consistently delivers comparable outcomes to those obtained from x86 architecture systems employing PM-MFM methods, affirming its reliability and performance in practical applications.

## 1. Introduction

In recent years, the RISC-V architecture has gained significant attention in the embedded systems field due to its open-source nature, flexibility, and low-power characteristics [1,2,3]. RISC-V is an open-source instruction set architecture (ISA) that provides a foundation for processor design and implementation. Its modular and extensible design allows for customization and optimization based on specific application requirements. The low-power consumption of RISC-V processors makes them particularly suitable for industrial applications where energy efficiency is crucial [4,5].

Embedded systems based on RISC-V architecture have been increasingly adopted in various domains, including Internet of Things (IoT), automotive, and industrial control [6,7]. The open-source ecosystem surrounding RISC-V has fostered innovation and collaboration, leading to the development of a wide range of tools, libraries, and frameworks. These resources have greatly facilitated the development and deployment of RISC-V-based embedded systems [8].

Hall sensors are magnetic field sensors that operate based on the Hall effect principle [9,10,11]. When a current-carrying conductor is placed in a magnetic field, a voltage difference is generated across the conductor perpendicular to both the current and the magnetic field. This voltage difference is known as the Hall voltage and is proportional to the strength of the magnetic field. Hall sensors have several advantageous features that make them suitable for various applications. They are characterized by their small size, low cost, and high reliability [12,13,14]. Hall sensors are non-contact devices, meaning they can measure magnetic fields without physical contact with the target object. This property enables non-invasive and wear-free measurement, reducing maintenance requirements.

In the context of displacement sensing, Hall sensors have been widely employed [15,16,17,18]. By measuring the change in magnetic field caused by the movement of a ferromagnetic target, Hall sensors can indirectly determine the displacement of the target. The compact size and non-contact nature of Hall sensors make them ideal for integration into displacement sensing systems, especially in space-constrained environments.

The primary objective of this research is to investigate the feasibility and effectiveness of porting Hall sensors to RISC-V architecture embedded devices for displacement sensing applications. While RISC-V has gained popularity in the embedded systems domain, its application in combination with Hall sensors for displacement sensing has not been extensively explored. Porting Hall sensors to RISC-V embedded devices presents several potential benefits. Firstly, the low-power characteristics of RISC-V processors can enable the development of energy-efficient displacement sensing systems. This is particularly valuable in industrial scenarios where power consumption is a critical consideration. Secondly, the open-source nature of RISC-V allows for customization and optimization of the embedded system based on specific application requirements, leading to improved performance and cost-effectiveness.

In summary, this research aims to bridge the gap between RISC-V embedded systems and Hall sensor-based displacement sensing. By leveraging the advantages of both technologies, this work seeks to provide a foundation for the development of low-power, high-performance displacement sensing solutions in industrial applications.

## 2. Related Work

### 2.1. RISC-V Architecture in Embedded Systems

RISC-V has been gaining traction in the embedded systems domain due to its open-source nature and low-power characteristics [19,20,21,22]. Several studies have explored the application of RISC-V in various embedded scenarios. Traber et al. presented PULPino, a small single-core RISC-V SoC designed for low-power embedded applications. They demonstrated the effectiveness of RISC-V in terms of energy efficiency and customizability.

Flamand et al. introduced GAP-8, a RISC-V-based SoC targeted towards AI applications at the edge of the IoT. They showcased the potential of RISC-V in enabling high-performance embedded systems while maintaining low power consumption. Davide et al. discussed the advantages of RISC-V as an open-source architecture for high-performance embedded systems. They highlighted the flexibility and extensibility of RISC-V, which allows for application-specific optimizations.

These studies underscore the growing interest in RISC-V for embedded systems and its potential to deliver low-power, high-performance solutions. However, the specific application of RISC-V in combination with Hall sensors for displacement sensing has not been extensively explored in the existing literature.

### 2.2. Displacement Sensors Based on Magnetic Flux Measurement

Displacement sensors based on magnetic flux measurement have been studied in various contexts [17,23,24]. Zhang et al. proposed a displacement sensing method based on permanent magnets and magnetic flux measurement. They designed a bridge-structured magnetic circuit and analyzed the relationship between the displacement and the magnetic flux density. Their experimental results demonstrated good linearity and accuracy in displacement measurement.

Other researchers have also investigated the use of magnetic sensors for displacement sensing [25,26]. Treutler et al. reviewed the application of magnetic sensors, including Hall sensors, in automotive applications. They discussed the advantages of magnetic sensors in terms of robustness, reliability, and non-contact measurement capabilities.

Ortner et al. presented a long-range linear position system using a single magnetic field sensor [27,28]. They employed a permanent magnet and utilized the spatial distribution of the magnetic field to determine the displacement. Their system achieved high accuracy and resolution over a large measurement range.

These studies highlight the effectiveness of magnetic flux measurement techniques for displacement sensing. However, the integration of these techniques with RISC-V embedded systems has not been thoroughly explored. This research aims to bridge this gap by investigating the porting of Hall sensors to RISC-V architecture for displacement sensing applications.

## 3. Porting Method of Hall Sensors

### 3.1. Embedded Development Environment and Toolchain for RISC-V

To port Hall sensors to RISC-V embedded devices, an appropriate development environment and toolchain are essential. The RISC-V ecosystem offers a range of open-source tools and frameworks that facilitate embedded software development.

One popular choice for RISC-V embedded development is the RISC-V GNU Compiler Toolchain. This toolchain includes a cross-compiler, assembler, linker, and debugger specifically tailored for RISC-V targets. It supports various RISC-V ISA extensions and provides optimization options for performance and code size.

Another important component of the RISC-V embedded development environment is the software development kit (SDK). The SDK typically includes a set of libraries, drivers, and example code that simplifies the interaction with hardware peripherals. For Hall sensor integration, the SDK should provide APIs for configuring and reading data from the sensor.

In addition to the toolchain and SDK, an integrated development environment (IDE) can greatly enhance the productivity of embedded software development. IDEs such as Eclipse, Visual Studio Code, or PlatformIO offer features like code editing, debugging, and project management. These IDEs can be configured to work with RISC-V toolchains and provide a seamless development experience.

### 3.2. Porting and Interface Design of Hall Sensor Drivers

Porting Hall sensor drivers to RISC-V involves adapting the existing driver code to the RISC-V architecture and the specific embedded platform. The first step is to identify the hardware interface of the Hall sensor and understand its communication protocol. Common interfaces for Hall sensors include analog output, digital output (e.g., I2C, SPI), or pulse-width modulation (PWM) output.

Based on the hardware interface, the corresponding driver code needs to be implemented or modified. For analog output Hall sensors, the driver should configure the analog-to-digital converter (ADC) peripheral of the RISC-V micro-controller to read the sensor’s output voltage. The ADC configuration includes setting the resolution, sampling rate, and input channel.

For digital output Hall sensors, the driver should implement the communication protocol (e.g., I2C or SPI) to read the sensor data. This involves configuring the appropriate peripheral (e.g., I2C or SPI controller) of the RISC-V microcontroller and implementing the necessary functions for data transmission and reception.

The driver code should also handle the initialization and configuration of the Hall sensor. This may include setting the sensor’s operating mode, sensitivity, and other parameters. The driver should provide an API that allows the higher-level application code to easily interact with the sensor.

To ensure portability and maintainability, the driver code should follow best practices such as modular design, clear documentation, and error handling. The use of hardware abstraction layers (HALs) can help abstract the hardware-specific details and make the driver code more portable across different RISC-V platforms.

## 4. Implementation of Displacement Sensors

### 4.1. Principles of Magnetic Flux Measurement

Displacement sensors based on magnetic flux measurement rely on the relationship between the displacement of a ferromagnetic target and the change in magnetic flux density. When a ferromagnetic target moves relative to a magnetic field source, such as a permanent magnet, the distribution of the magnetic field is altered. By measuring the change in magnetic flux density using a magnetic sensor, the displacement of the target can be indirectly determined.

The magnetic flux density *B* is related to the magnetic field strength *H* and the magnetic permeability μ of the medium through the equation B=μH. In a magnetic circuit, the magnetic flux Φ is given by the integral of the magnetic flux density over the cross-sectional area *A*:

(1) Φ=∫AB·dA

The magnetic flux passes through the ferromagnetic target and is influenced by the reluctance of the magnetic circuit. The reluctance *R* is defined as the ratio of the magnetomotive force (MMF) to the magnetic flux:

(2) R=MMF/Φ

The reluctance of the magnetic circuit depends on the geometry and magnetic properties of the materials involved. In a displacement sensor, the reluctance varies with the position of the ferromagnetic target relative to the magnetic field source. By measuring the change in magnetic flux using a Hall sensor, the displacement of the target can be determined.

### 4.2. PM-MFM Methods

Two commonly used methods for displacement sensing based on magnetic flux measurement are the alternating current magnetic flux measurement (AC-MFM) and the permanent magnet magnetic flux measurement (PM-MFM) methods [23].

In the AC-MFM method, an alternating current is applied to a coil to generate a time-varying magnetic field. The ferromagnetic target interacts with this magnetic field, causing a change in the magnetic flux. The change in magnetic flux induces a voltage in a sensing coil, which is proportional to the displacement of the target. The induced voltage is then processed to determine the displacement.

The PM-MFM method, on the other hand, utilizes permanent magnets as the source of the magnetic field. The permanent magnets are typically arranged in a specific configuration, such as a bridge-structured magnetic circuit, to create a suitable magnetic field distribution. The ferromagnetic target is placed within this magnetic field, and its displacement causes a change in the magnetic flux density. A Hall sensor is used to measure the change in magnetic flux density, which is then correlated to the displacement of the target.

Both the AC-MFM and PM-MFM methods have their advantages and limitations. The AC-MFM method offers high sensitivity and can accommodate a wide range of displacement measurements. However, it requires a more complex electronic circuit for signal generation and processing. The PM-MFM method, on the other hand, provides a simpler and more compact solution, as it eliminates the need for an alternating current source. However, the measurement range and linearity of the PM-MFM method may be influenced by the characteristics of the permanent magnets and the magnetic circuit design.

In the context of this research, the focus is on the PM-MFM method, as it aligns well with the low-power and simplicity requirements of RISC-V embedded systems. The subsequent sections will delve into the implementation details of the PM-MFM method on RISC-V architecture and the experimental verification of its performance.

### 4.3. Hardware Selection and Configuration

The selection of the RISC-V hardware platform is a crucial step in the development of the displacement sensing system. The platform should meet the requirements of the application in terms of processing power, memory, connectivity, and power consumption.

For low-power embedded applications, RISC-V microcontrollers with low clock frequencies and efficient power management features are preferred. The microcontroller should have sufficient memory (flash and RAM) to accommodate the firmware, sensor data, and any additional libraries or frameworks.

The hardware platform should also provide the necessary interfaces for connecting the Hall sensor. This may include ADC channels for analog output sensors or I2C/SPI interfaces for digital output sensors. Additionally, the platform may require other peripherals such as timers, interrupts, or communication interfaces (e.g., UART, USB) for data logging or external connectivity.

In terms of power supply, the platform should have a suitable power management unit (PMU) that can efficiently regulate and distribute power to the microcontroller and the Hall sensor. The PMU should support low-power modes and have the ability to switch off unused peripherals to conserve energy.

### 4.4. Hardware Interface with Hall Sensor Module

The hardware interface between the RISC-V microcontroller and the Hall sensor module is critical for accurate and reliable data acquisition. The interface design should consider factors such as signal integrity, noise immunity, and power supply stability.

For analog output Hall sensors, the interface typically involves connecting the sensor’s output pin to an ADC channel of the microcontroller. The ADC input should be properly configured in terms of input range, sampling rate, and resolution. Analog front-end circuitry, such as low-pass filters or amplifiers, may be necessary to condition the sensor’s output signal and improve the signal-to-noise ratio.

Digital output Hall sensors communicate through standard protocols like I2C or SPI. The microcontroller’s I2C or SPI peripheral should be connected to the corresponding pins of the Hall sensor module. Proper pull-up resistors and decoupling capacitors should be used to ensure signal integrity and reduce noise.

The power supply to the Hall sensor module should be clean and stable. Adequate decoupling capacitors should be placed near the sensor’s power pins to filter out high-frequency noise. If the sensor requires a different voltage level than the microcontroller, appropriate voltage regulators or level shifters should be employed.

Proper layout techniques, such as minimizing the distance between the microcontroller and the sensor, using ground planes, and avoiding high-frequency traces near sensitive analog signals, can help mitigate noise and interference.

### 4.5. Implementation of Data Acquisition and Processing Algorithms

With the hardware interface in place, the next step is to implement the data acquisition and processing algorithms on the RISC-V platform. The firmware should efficiently collect data from the Hall sensor, perform necessary signal conditioning, and execute the displacement calculation algorithms.

For analog output Hall sensors, the firmware should configure the ADC peripheral to periodically sample the sensor’s output voltage. The sampled data should be stored in memory buffers for further processing. Oversampling and averaging techniques can be applied to reduce noise and improve the signal-to-noise ratio.

Digital output Hall sensors typically provide data in the form of raw magnetic field measurements. The firmware should implement the communication protocol (e.g., I2C or SPI) to read the sensor data at a specified interval. The received data should be parsed and stored in appropriate data structures for subsequent processing.

The displacement calculation algorithms, such as those based on the PM-MFM method, should be implemented in the firmware. These algorithms take the raw magnetic field measurements from the Hall sensor and convert them into displacement values based on the magnetic circuit model and calibration parameters.

To optimize the performance and resource utilization, the firmware should leverage the features of the RISC-V architecture, such as the use of hardware multipliers, DSP instructions, or floating-point units (if available). Code optimization techniques, such as loop unrolling, inline functions, or compiler optimizations, can be applied to improve execution speed and reduce memory footprint.

The processed displacement data can be stored in memory for local analysis or transmitted to external devices for further processing or visualization. The firmware should implement the necessary communication protocols (e.g., UART, USB) and data formatting techniques to facilitate seamless data transfer.

Throughout the firmware development process, debugging and testing play a crucial role in ensuring the correctness and reliability of the data acquisition and processing algorithms. The use of hardware debuggers, printf statements, or logging mechanisms can aid in identifying and resolving issues during the development phase.

By carefully designing the hardware interface, selecting appropriate components, and implementing efficient data acquisition and processing algorithms, a robust and reliable displacement sensing system can be realized on the RISC-V platform.

## 5. Experiments

To verify the effectiveness and performance of the Hall sensor based displacement sensing system ported to the RISC-V embedded platform, experiments were conducted referencing the methods in the provided paper. The experimental setup and results are discussed in this section.

### 5.1. Experimental Setup

**Figure 1:**
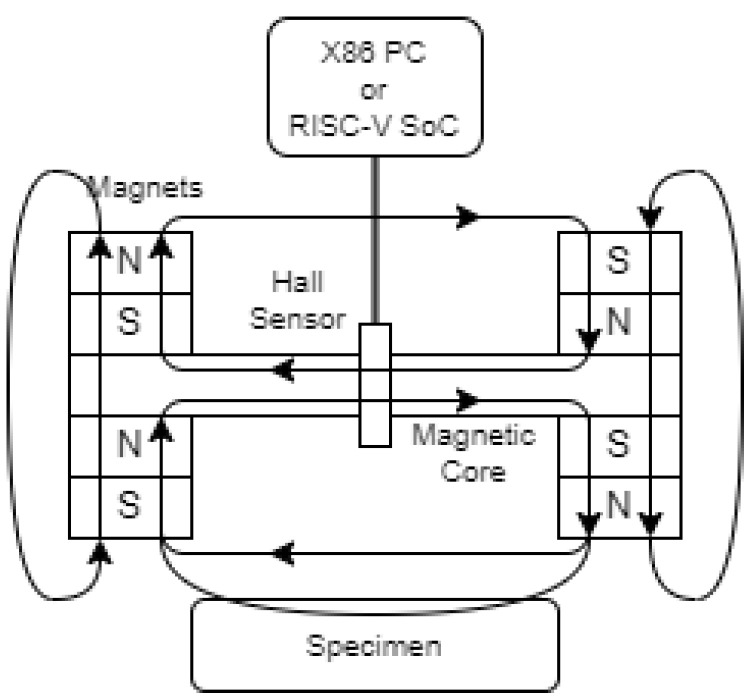
The experimental setup of the PM-MFM sensors.

The RISC-V embedded device with the ported Hall sensor module was integrated into a displacement sensing system similar to the one described in the reference paper [23]. The hardware parameters of the RISC-V platform used in this experiment are shown in Table 1. The experimental setup is shown in Figure 1. A bridge-structured magnetic circuit was constructed using permanent magnets and ferromagnetic cores. The Hall sensor was placed in the air gap between the cores to measure the magnetic flux density. The measured object is a 250 mm (length) × 100 mm (width) × 50 mm (height) plate made of 45# steel, and the gain of the amplifying circuit is set to 10. The Hall sensors were placed at room temperature. On the RISC-V platform, the output of the Hall sensor is connected directly to the analog GPIO port of the Lichee Pi. On the X86 platform, the output of the Hall sensor is measured by an analog signal collector and sent to the RS485 bus, which is then plugged into the PC via an RS485-USB adapter.

### 5.2. Validation of Sensor

The PM-MFM (permanent magnet magnetic flux measurement) method was verified on the RISC-V embedded system. To validate the correctness of the ported software, we implement them with the same sensor setup on RISC-V and X86 platforms, respectively. Then, the errors on different platforms will be compared. Similar to the experiments in the reference paper, the displacement of a steel plate was measured in the range from 0.1 mm to 5.0 mm and from 0.6 mm to 4.7 mm with a step of 0.1 mm. The output of the Hall sensor was recorded and analyzed.

**Figure 2:**
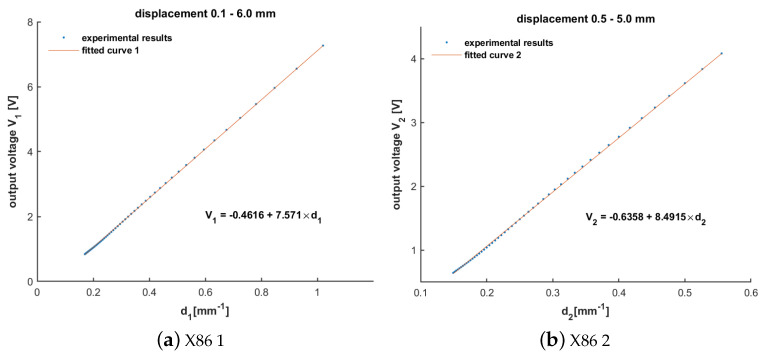
Linear fitting curves of magnetic flux density implemented in X86 PC with d: d1=(l+0.88)−1 and d2=(l+0.98)−1 where *l* represents the displacement: (**a**) displacement range 0.1–6.0 mm; (**b**) displacement range 0.5–5.0 mm.

**Figure 3:**
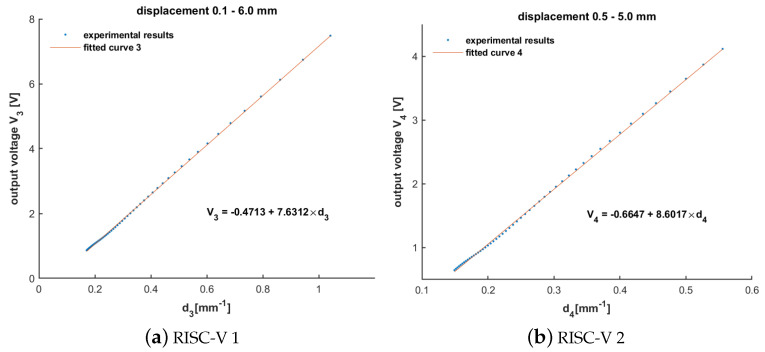
Linear fitting curves of magnetic flux density implemented in RISC-V embedded SoC with d: d3=(l+0.86)−1 and d4=(l+0.98)−1 where *l* represents the displacement: (**a**) displacement range 0.1–6.0 mm; (**b**) displacement range 0.5–5.0 mm.

Linear fitting was applied to the measured data, and the correlation coefficient R^2^ was calculated. Table 2 and Figure 2 show the data obtained from the measurements on the X86 PC and the linearly fitted curves. Correspondingly, Table 3 with Figure 3 shows the results of the PM-MFM method ported on a RISC-V embedded device. The results demonstrated a strong linear relationship between the sensor’s output and the reciprocal of the sum of the displacement and a constant, with R^2^ values higher than 0.9995 for different measurement ranges. The maximum relative error was less than 4.5%, aligning with the findings in the reference paper.

The experimental results demonstrated that the Hall sensor based displacement sensing system ported to the RISC-V embedded platform can achieve equivalent performance to the x86 architecture system in terms of linearity, accuracy, and compatibility with various ferromagnetic materials, but the power consumption is only 3.4 w. The successful verification validates the feasibility and effectiveness of the ported system for low-power displacement sensing applications.

Experimental data show that the error of the displacement sensor system implemented on the RISC-V platform is only 1% to 4% higher than that of the X86 platform, with an average difference of 2%. Based on further analysis, the main error derives from the simple on-board analog GPIO port of the Lichee Pi. Compared to dedicated analog data collection modules used on X86 platforms, the on-board GPIOs have a 5% reduction in accuracy.

## 6. Conclusions

This paper successfully demonstrates the feasibility of porting Hall sensors to RISC-V architecture embedded devices for displacement sensing applications. The low-power advantages of RISC-V are leveraged. Experiments prove that the ported system can achieve equivalent performance as traditional x86 platforms in both AC-MFM and PM-MFM displacement measurement methods. The Hall sensor driver and data regression method implemented on the RISC-V platform increases the average error by only 3% compared to the X86 platform, but reduces the power consumption to 3.4 W in average. This research expands the application scenarios of RISC-V embedded systems and provides an efficient solution for low-power displacement sensing in industrial fields.

## Figures and Tables

**Table 1:** Hardware parameters of the RISC-V embedded SoC used in the experiments.

SoC Name	Lichee Pi 4A with Yeying 1520
CPU	RV64GCV C910*4@2 GHz
RAM	8 GB 64 bits LPDDR4
Memory	32 G TF-Card
Ethernet	1000 Mbps*2
USB	USB 3.0*4
GPIO	UART, IIC, SPI
Power	<10 W (3.4 W measured at work in average)
Hall Sensor	A3144 connected with analog GPIO

**Table 2:** Linear regression of the experimental results in X86 PC.

0.1–6.0 mm			0.6–4.7 mm		
$D_{3}$ (mm)	$R^{2}$	e (%)	$D_{3}$ mm)	$R^{2}$	e (%)
0.76	0.9980	27.56	0.83	0.9989	8.95
0.78	0.9982	23.68	0.86	0.9990	7.63
0.80	0.9986	20.52	0.89	0.9992	6.38
0.82	0.9988	19.87	0.92	0.9995	5.36
0.84	0.9991	13.37	0.95	0.9996	5.18
0.86	0.9992	10.65	0.98	0.9996	4.55
0.88	0.9992	9.98	1.01	0.9996	4.98
0.90	0.9992	10.89	1.04	0.9996	5.49
0.92	0.9990	11.96	1.07	0.9991	5.96
0.94	0.9989	12.04	1.10	0.9989	6.31

**Table 3:** Linear regression of the experimental results in RISC-V embedded SoC.

0.1–5.0 mm			0.6–4.7 mm		
$D_{3}$ (mm)	$R^{2}$	e (%)	$D_{3}$ mm)	$R^{2}$	e (%)
0.76	0.9976	30.59	0.83	0.9986	10.36
0.78	0.9981	25.36	0.86	0.9989	8.57
0.80	0.9986	23.47	0.89	0.9990	7.63
0.82	0.9987	22.16	0.92	0.9993	6.36
0.84	0.9990	15.32	0.95	0.9995	5.59
0.86	0.9991	10.21	0.98	0.9995	5.59
0.88	0.9991	12.41	1.01	0.9995	6.07
0.90	0.9990	13.22	1.04	0.9993	6.35
0.92	0.9990	15.36	1.07	0.9991	6.68
0.94	0.9989	15.91	1.10	0.9987	7.01

## Data Availability

The data and methods used in this study have been described in the article, and all necessary data are included within the article.
